# Factors affecting the occurrence of maxillary sinus fungus ball

**DOI:** 10.3389/fsurg.2024.1491155

**Published:** 2024-11-01

**Authors:** Hye-Bin Jang, Dong Hoon Lee, Sang Chul Lim

**Affiliations:** Department of Otolaryngology–Head and Neck Surgery, Chonnam National University Medical School & Hwasun Hospital, Hwasun, Republic of Korea

**Keywords:** maxillary sinus, mycoses, computed tomography, maxillary sinusitis, trauma, dental implants

## Abstract

**Objective:**

We identified patients who initially did not have a maxillary sinus fungus ball on computed tomography (CT) but developed it on a subsequent CT scan. We assessed potential risk factors for developing a maxillary sinus fungus ball between the two scans.

**Patients and methods:**

This study included 35 patients with 38 lesions who initially had no maxillary sinus fungus balls on CT but were later diagnosed with the condition and underwent surgery.

**Results:**

We analyzed 38 lesions in 35 patients, each of whom had normal CT scan results initially but later developed a maxillary sinus fungus ball. No specific risk factors for developing a maxillary sinus fungus ball were identified. However, when compared to the maxillary sinusitis group, facial trauma and dental implant surgery appeared to be associated with maxillary sinus fungus ball formation (*p* < 0.05).

**Conclusion:**

We investigated factors influencing the development of maxillary sinus fungus balls that were absent in previous CT scans and found no significant risk factors. Nonetheless, relative to the maxillary sinusitis (control) group, the maxillary sinus fungus ball group tended to have more previous facial trauma and dental implant surgery.

## Introduction

A paranasal sinus fungus ball is a form of non-invasive fungal sinusitis primarily caused by *Aspergillus* in the maxillary sinus (1–8). The pathogenesis of fungus ball development remains unclear, though anatomical variations and dental treatments are being investigated as potential risk factors (1–8). However, owing to the unknown onset timing of maxillary sinus fungus ball formation, identifying specific risk factors is challenging. Consequently, we identified patients who initially did not have maxillary sinus fungus balls on computed tomography (CT) but developed such lesions on subsequent CT scans. Between these CT scans, we evaluated the presence of risk factors for maxillary sinus fungus ball development. Additionally, we compared the characteristics of maxillary sinus fungus ball cases with those of a control group of patients who underwent endoscopic sinus surgery for maxillary sinusitis.

### Patients and methods

This study was conducted following approval by the Institutional Review Board of Chonnam National University Hwasun Hospital. The study included 35 patients with 38 lesions who initially had no maxillary sinus fungus balls on CT but were subsequently diagnosed with the condition and underwent surgery. All cases underwent an initial CT scan when no maxillary sinus fungus ball was present and a subsequent CT scan before surgery for maxillary sinus fungus ball, resulting in at least two CT scans per case. That is, we first selected patients who had undergone maxillary sinus fungus balls and then checked whether they had a previous normal CT scan. Patients had previously undergone CT scans for health screening purposes, facial or head trauma, or symptoms such as headache. We excluded patients with maxillary sinus fungus balls detected by CT before the diagnosis of maxillary sinusitis and those who had no previous CT scans available.

To investigate the causes of fungus ball development over time in the maxillary sinus where no fungus ball was initially present, factors such as facial trauma, dental cavity treatment, dental implant surgery, septal deviation, Haller cells, concha bullosa, and previous nasal surgery were analyzed. All patients underwent endoscopic sinus surgery, and all maxillary sinus lesions were confirmed histopathologically. Fisher's exact test, chi-square test, and Mann-Whitney *U* test were used to determine statistical significance. R version 4.2.3 (R Foundation for Statistical Computing, Vienna, Austria) was used for all statistical analyses. Statistical significance was defined as a *p* < 0.05.

## Results

In this study, 38 lesions in 35 patients who had normal CT scan results but later developed maxillary sinus fungus balls were analyzed. The mean period between the normal CT scan and the diagnostic CT for maxillary sinus fungus ball formation was 89.7 ± 49.6 months (range, 13–219 months). To investigate the causes of fungus ball development over time in the maxillary sinus where no fungus ball was initially present, factors such as facial trauma, dental cavity treatment, dental implant surgery, septal deviation, Haller cells, concha bullosa, and previous nasal surgery were analyzed. Unfortunately, none of the results were statistically significant.

To analyze the characteristics associated with maxillary sinus fungus ball formation, a control group consisting of patients who underwent surgery for maxillary sinusitis with similar demographic characteristics such as age, sex, and site of occurrence, was created. Clinical findings associated with both maxillary sinus fungus ball formation and maxillary sinusitis are summarized in Table 1.

**Table 1:** Clinical findings of patients with maxillary sinus fungus ball and maxillary sinusitis.

	Maxillary sinus fungus ball (Total = 38)	Maxillary sinusitis (Total = 40)	*p*-value
Sex (male: female)	16:19	17:18	
Age (years)	40–91 (67.8 ± 12.8)	37–85 (61.0 ± 12.1)	
Past medical history			
Hypertension	18	13	
Diabetes	12	7	
Malignancy	7	4	
Most common symptoms			
Nasal obstruction	7	3	
Accidental diagnosis	6	7	
Rhinorrhea	5	10	
Facial pain	5	2	
Postnasal drip syndrome	3	7	
Duration of symptoms (months)	0.3–24 (6.1 ± 6.5)	0.1–360 (20.1 ± 62.7)	>0.05
Location (right: left: bilateral)	25:7:3	13:17:5	<0.05
Smoking history	10	13	>0.05
Facial trauma history	15	5	<0.05
Dental cavity treatment	8	8	>0.05
Dental implant surgery	11	4	<0.05
Anatomical variation			
Septal deviation	9	5	>0.05
Haller cells	9	7	>0.05
Concha bullosa	4	5	>0.05
History of nose surgery	6	1	>0.05
Anesthesia (general: local)	9:26	2:33	>0.05
Histopathological result	*Aspergillus* (*n* = 38)	Chronic sinusitis (*n* = 40)	

The 38 lesions in 35 patients with maxillary sinus fungus balls included 16 men and 19 women. Their mean age was 67.8 ± 12.8 years (range, 40–91 years). In past medical history, 18 people had hypertension, 12 had diabetes, and 7 had cancer. The most common symptoms associated with maxillary sinus fungus ball formation were nasal obstruction (*n* = 7, 20.0%), incidental discovery during imaging tests (*n* = 6, 17.1%), rhinorrhea (*n* = 5, 14.3%), facial pain (*n* = 5, 14.3%), and postnasal drip syndrome (*n* = 3, 8.6%). The duration of symptoms was 6.1 ± 6.5 months (range, 0.3–24 months). Maxillary sinus fungus balls occurred in the right maxillary sinus in 25 patients, the left maxillary sinus in seven patients, and both maxillary sinuses in three patients. In the maxillary sinus fungus ball group, there were ten smokers (28.6%) with an average of 32.3 ± 17.1 pack-years.

There were 15 cases involving facial trauma, including 13 patients with a history of facial trauma and two patients with bilateral maxillary sinus fungus balls. There were eight cases involving dental cavity treatment, including seven patients treated for cavities in the ipsilateral maxillary teeth and one patient treated on both sides. There were 11 patients who received dental implant surgery on the ipsilateral maxillary teeth. There were nine patients with septal deviation where the maxillary sinus fungus ball occurred on the same side. Haller cells were found in nine cases, including one bilateral case. Concha bullosa was found in four cases, including one bilateral case. There were six patients with a history of nasal surgery, including endoscopic sinus surgery or septoplasty. All 35 patients underwent endoscopic sinus surgery. Among them, 26 patients underwent surgery under local anesthesia, and nine patients under general anesthesia. According to histopathology results, all patients were infected with *Aspergillus*.

The 40 lesions in 35 patients with maxillary sinusitis included 17 males and 18 females. Their mean age was 61.0 ± 12.1 years (range, 37–85 years). In past medical history, 13 people had hypertension, 7 had diabetes, and 4 had cancer. The most common symptoms of chronic maxillary sinusitis were rhinorrhea (*n* = 10, 28.8%), incidental discovery during imaging tests (*n* = 7, 20.0%), postnasal drip syndrome (*n* = 7, 20.0%), nasal obstruction (*n* = 3, 8.6%), and facial pain (*n* = 2, 5.7%). The duration of symptoms was 20.1 ± 62.7 months (range, 0.1–360 months). Maxillary sinusitis occurred in the right maxillary sinus in 13 patients, the left maxillary sinus in 17 patients, and both maxillary sinuses in five patients. In the maxillary sinusitis group, there were 13 smokers (37.1%), with an average of 36.7 ± 32.4 pack-years.

There were five cases with a history of facial trauma, eight cases treated for cavities in the ipsilateral maxillary teeth, and four patients who received dental implant surgery on the ipsilateral maxillary teeth. There were five patients with septal deviation where the maxillary sinusitis occurred on the same side. Haller cells were found in seven cases, including one bilateral case. Concha bullosa was found in five cases, including one bilateral case. One patient had a history of endoscopic sinus surgery. All 35 patients underwent endoscopic sinus surgery, with 33 under local anesthesia and two under general anesthesia. According to histopathological results, all control patients had chronic maxillary sinusitis, and no fungus balls were found.

Among cases with maxillary sinus fungal balls—relative to the maxillary sinusitis (control) group—facial trauma and dental implant surgery were found to be significantly associated with the occurrence of maxillary sinus fungus balls (*p* < 0.05). However, the main symptoms, smoking history, dental cavity treatment, septal deviation, Haller cells, concha bullosa, and previous nasal surgery were not statistically significant factors. No major complications resulted from the surgical interventions.

## Discussion

In this study, we aimed to identify the factors influencing the occurrence of new-onset maxillary sinus fungus balls in patients with maxillary sinusitis. Various factors, including facial trauma, dental treatment, anatomical variations, and nasal surgery, were analyzed, but none showed statistical significance.

**Figure 1:**
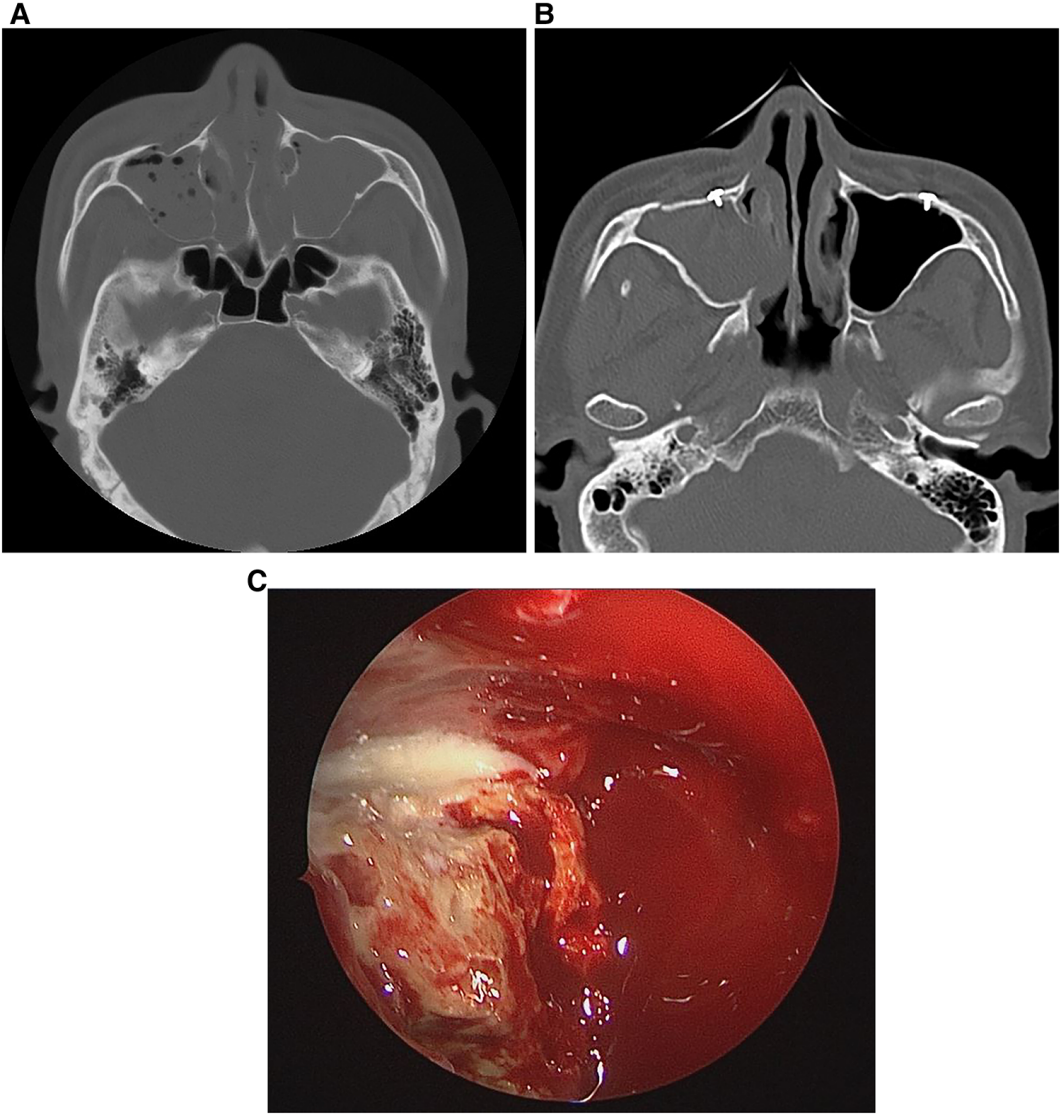
A 54-year-old man with **(A)** bilateral maxillary sinus fractures due to trauma 18 years prior with fluid and air observed in both maxillary sinuses from bleeding. **(B)** A month before visiting the hospital, the patient complained of right-sided nasal discharge; CT showed haziness and calcification in the right maxillary sinus, suggesting a fungus ball. **(C)** Intraoperative photograph showing the maxillary sinus fungus ball.

Additionally, this study compared maxillary sinus fungus ball cases with a control group that underwent surgery for maxillary sinusitis. Facial trauma and dental implant surgery were found to be statistically significant in the occurrence of maxillary sinus fungus balls (2–5, 7–9). Previous studies did not specifically mention facial trauma, but our results demonstrated its significance in first occurrences of maxillary sinus fungus balls (Figure 1).

There have been reports of a relationship between dental treatment and the occurrence of maxillary sinus fungus balls (2–5, 7, 8). In this study, dental implant surgery was found to be statistically significant, while dental cavity treatment was not.

Reports also suggest relationships between anatomical variations of the paranasal sinuses and the occurrence of maxillary sinus fungus balls (2–5). However, in this study, anatomical variations, including septal deviation, Haller cells, and concha bullosa, were not associated with the occurrence of maxillary sinus fungus balls, consistent with previous findings (6).

In this study, maxillary sinus fungus balls were found to occur more frequently on the right side than maxillary sinusitis. This discrepancy may be due to the small sample size or the higher incidence of facial trauma or dental implant procedures on the right side. Therefore, to confirm this finding, analysis of a larger patient cohort is needed.

The treatment of choice for maxillary sinus fungus balls is surgical removal (1, 3, 8, 10). The prognosis after appropriate surgical removal is excellent (10). In this study, all patients with maxillary sinus fungus balls underwent endoscopic sinus surgery, with no recurrences or surgical complications observed.

## Conclusion

We analyzed factors affecting the occurrence of maxillary sinus fungus ball, which was not present in the past, but no significant factors were found. Compared to the maxillary sinusitis group, the maxillary sinus fungus ball group tended to have more facial trauma and dental implant surgery.

## Data Availability

The raw data supporting the conclusions of this article will be made available by the authors, without undue reservation.
